# Morphine for Quinine

**Published:** 1885-10-20

**Authors:** 


MORPHINE FOR QUININE.—So frequent are the fatal mistakes, by druggists, of putting up morphine for quinine that really something should be done by way of protection. The last mistake of which we read was by a physician who, being drunk, gave 10 grains of morphine to three individuals at the same time and in the same family, killing every one! And yet in almost every community there is a drinking doctor. He is usually very popular, and is esteemed a genius in medicine. In fact, there are many who will say, "I had rather have him drunk than any other doctor sober."

